# Alumni Association of Chicago Medical College

**Published:** 1882-05

**Authors:** 


### ARTICLE XI.

### ALUMNI ASSOCIATION OF THE CHICAGO MEDICAL COLLEGE.

The annual meeting of the Alumni Association of the Chicago Medical College took place Tuesday, March 28, at 2 P. M. After an address by the President, Dr. D. A. K. Steele, and the Necrologist's report, by Dr. F. E. Waxham, the following prizes were distributed:

Senn prize, for best anatomical preparation, by a student of Chicago Medical College, one volume Surgery. Taken by E. Wing, M. D.

Prize for highest average scholarship for three years in Chicago Medical College. Taken by W. E. Morgan. E. Wing and E. L. Hollister followed closely on the college list.

Edwards' prize, for best scholarship in second year class, one set Wood's Library. Taken by N. S. Davis, Jr.

Alumni prize essay, for best Thesis. Taken by M. P. Hatfield, M. D. Subject: Prophylaxis and Treatment of Dyspepsia of Infants.

After music, the society went into executive session. The following list of officers was elected:

President—M. P. Hatfield, of Chicago.

First Vice President—A. J. Smith, of Indiana.

Second Vice President—Charles Goddard, of Illinois.

Necrologist—F. E. Waxham, of Chicago.

Secretary and Treasurer—H. T. Byford, of Chicago.

Dr. Roswell Park moved that the resolution of last year concerning the prize essay be continued. His motion was carried. Following is the resolution:

*Resolved*, That the Chicago Medical College Alumni Association set aside the sum of $50 as a prize for the best essay on either one of the subjects to be selected by a committee of three. Such essay must be of sufficient merit, preference being given to original work and observation. Essays are to be handed to the secretary of the association before March 1, 1883, accompanied by a sealed envelope containing some device or motto similar to the one on the manuscript. All competitors shall be graduates, and have paid the annual dues. Award will be made by the Permanent Committee of Prizes—Drs. N. S. Davis, H. A. Johnson and E. O. F. Roler.

---

ADVANCING THE STANDARD.—There is no doubting the fact that the standard of qualifications for graduation in the medical schools of this city (New York), has materially advanced within the past few years. The system of examination is more rigid than at any time heretofore, and great pains are taken to prevent the graduation of unqualified persons. Written examinations are the rule; the questions given are selected with remarkable care, and a given standard is strictly enforced by a direct vote of the faculty on the papers of each candidate.—MED. REC.

In these particulars, Chicago is not behind New York.

DEAFNESS IN SCHOOL CHILDREN.—At the request of the Commissioner of Education, Dr. Samuel Sexton has made some investigations regarding the prevalence of deafness among school children, its causes and prevention. He examined 570 pupils in the public and parochial schools of New York City. In seventy-six persons, or about thirteen per cent., there was greatly diminished hearing in one or both ears. In only one case had any deafness been suspected previously either by teacher or pupil.—MED. REC.

